# Cnidocyte discharge is regulated by light and opsin-mediated phototransduction

**DOI:** 10.1186/1741-7007-10-17

**Published:** 2012-03-05

**Authors:** David C Plachetzki, Caitlin R Fong, Todd H Oakley

**Affiliations:** 1 Center for Population Biology, University of California at Davis, One Shields Avenue, Davis CA, 95616, USA; 2 Department of Ecology, Evolution, and Marine Biology, University of California at Santa Barbara, Santa Barbara CA, 93106, USA

**Keywords:** cnidocyte, stenotele, Cnidaria, opsin, phototransduction, arrestin, cyclic nucleotide gated ion channel.

## Abstract

**Background:**

Cnidocytes, the eponymous cell type of the Cnidaria, facilitate both sensory and secretory functions and are among the most complex animal cell types known. In addition to their structural complexity, cnidocytes display complex sensory attributes, integrating both chemical and mechanical cues from the environment into their discharge behavior. Despite more than a century of work aimed at understanding the sensory biology of cnidocytes, the specific sensory receptor genes that regulate their function remain unknown.

**Results:**

Here we report that light also regulates cnidocyte function. We show that non-cnidocyte neurons located in battery complexes of the freshwater polyp *Hydra magnipapillata *specifically express opsin, cyclic nucleotide gated (CNG) ion channel and arrestin, which are all known components of bilaterian phototransduction cascades. We infer from behavioral trials that different light intensities elicit significant effects on cnidocyte discharge propensity. Harpoon-like stenotele cnidocytes show a pronounced diminution of discharge behavior under bright light conditions as compared to dim light. Further, we show that suppression of firing by bright light is ablated by cis-diltiazem, a specific inhibitor of CNG ion channels.

**Conclusions:**

Our results implicate an ancient opsin-mediated phototransduction pathway and a previously unknown layer of sensory complexity in the control of cnidocyte discharge. These findings also suggest a molecular mechanism for the regulation of other cnidarian behaviors that involve both photosensitivity and cnidocyte function, including diurnal feeding repertoires and/or substrate-based locomotion. More broadly, our findings highlight one novel, non-visual function for opsin-mediated phototransduction in a cnidarian, the origins of which might have preceded the evolution of cnidarian eyes.

## Background

Animal sensory systems provide a useful model for understanding the origins and evolution of complex traits. Of particular interest are questions regarding (1) the ancestral composition of sensory signaling pathways, and (2) the ancestral function of such pathways. While a detailed understanding of the signaling pathways and cell types that function in the diversity of animal sensory systems is becoming increasingly common, most work in sensory molecular biology has been confined to an exceedingly small taxonomic sample of model bilaterian species. In order to gain insights into the evolutionary origins and ancestral functions of bilaterian sensory systems we must focus our attention on taxa such as the Cnidaria that represent an evolutionary sister group to bilaterians.

Perhaps the best understood animal sensory modality is photosensitivity. All known examples of visual perception in animals are accomplished, at the physiological level, by an opsin-mediated phototransduction cascade [1]. Animal phototransduction is a canonical G protein coupled receptor (GPCR) signaling pathway that results universally in a shift in the electro-chemical potential of photoreceptor neurons by the opening or closing of an ion channel [2,3]. Recent analyses of animal opsin phylogeny place the origin of animal phototransduction at the last common ancestor of the Cnidaria and Bilateria [4-6]. Numerous opsins are present in the two cnidarian genomes sequenced to date, *Hydra magnipapillata *and *Nematostella vectensis*, but paradoxically both of these taxa lack eyes or ocelli and are known to possess only dispersed, dermal photosensitivity [7-9]. The cnidarian phototransduction cascade is similar to the vertebrate 'ciliary' mode of phototransduction in that it utilizes a cyclic nucleotide gated (CNG) ion channel and other components of the vertebrate visual cycle [10-12]. Arrestins, which act to quench phototransduction by binding activated opsin [13] are another common feature of bilaterian phototransduction pathways [14-16], but their involvement in cnidarian phototransduction systems has yet to be examined.

**Figure 1:**
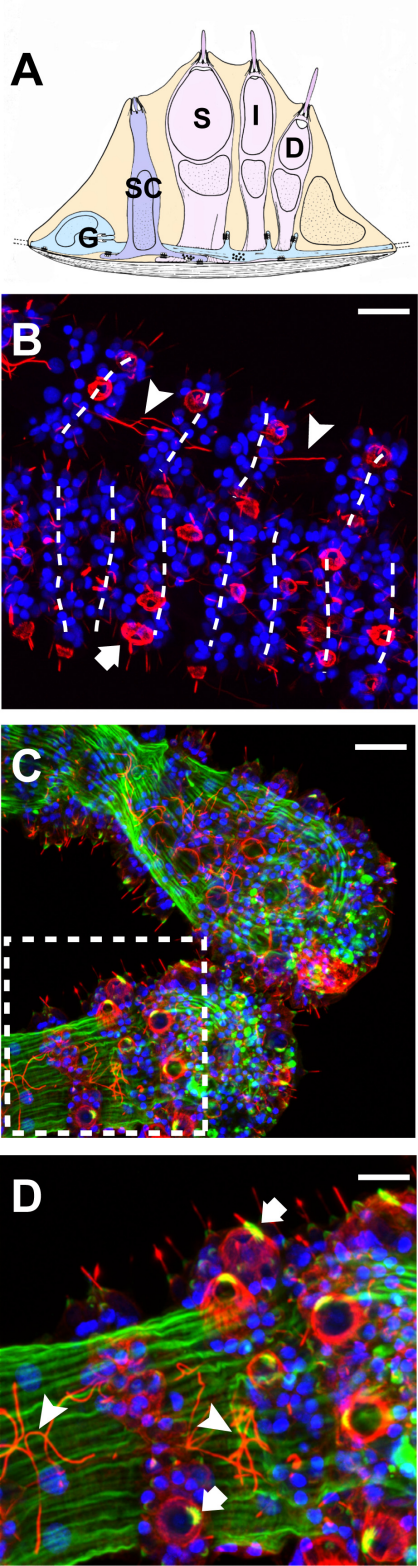
**Cnidocyte morphology and arrangement in *H. magnipapillata***. (**A**) A generalized schematic of the hydrozoan battery complex in cross-section. Stenotele cnidocytes (S) are flanked by isorhiza (I) and desmoneme (D) cnidocytes, as well as sensory cells (SC), which form synaptic connections with ganglion cells (G) that link each of the other cnidocyte cell types. (**B-D**) Confocal z-stacks of *H. magnipapillata *tentacle midsection (B) and tentacle bulbs (C and D). Neurons, including cnidocytes, are stained red (anti acetylated α-Tubulin) and nuclei are stained blue (DAPI). Musculature is shown in green (phalloidin) in (C and D). Axonal projections (arrow heads) link battery complexes that occur with regular periodicity along the length of the tentacle. Arrows indicate stenotele cnidocytes. (D) is an inset of (C). (A) Redrawn with permission from Westfall [36].

How is opsin-mediated phototransduction utilized in these eyeless cnidarian taxa? Cnidocytes and other cnidarian sensory neurons may provide some clues to this question. Cnidocytes are found only in the Cnidaria and are among the most complex cell types known in animals [17,18]. When properly stimulated, cnidocytes eject elaborate, energetically expensive, single-use, secretion products called cnidae that function in a range of organismal phenotypes including defense, prey capture, structure, and locomotion [18,19]. In hydrozoans, both cnidocytes and sensory neurons are found in specialized cellular consortiums called battery complexes [20] (Figure 1A). Such battery complexes consist of sets of functionally integrated cnidocytes that share common synaptic connections with both closely apposed sensory neurons and ganglion cells [21] (Figure 1A). Current evidence suggests that the cellular architecture of the hydrozoan battery complex functions to integrate sensory cues from the environment into the precise regulation of cnidocyte discharge [22-25].

While the genes that underlie the unique morphological and functional features of cnidocytes have been the focus of much enquiry [26-28], less attention has been aimed at the genetic bases for the complex sensory repertoires that regulate their function. Both chemical and mechanical sensory modalities have been shown to contribute to the regulation of cnidocyte discharge [24,29], but to date no sensory receptor gene has been linked to any of these behaviors in Cnidaria.

In addition to chemosensory and mechanosensory behaviors, cnidarians are known to display a range of photobehaviors that include diurnal migration [30], phototaxis [31], and contraction responses to light [7,32]. Because these behaviors are often associated with feeding or defense, two activities that involve cnidocytes, we hypothesized that light information from the environment might also play a role in regulating cnidocyte function. Here we show that photosensitivity acts to modulate cnidocyte discharge in the hydra *H. magnipapillata *and that this property is driven by an opsin-mediated phototransduction cascade. We report that opsin and other phototransduction components are expressed in cells that accompany harpoon-like stenotele cnidocytes in battery complexes. We further demonstrate that the propensity for cnidocyte discharge can be altered experimentally as a function of light intensity. Finally, the alteration of cnidocyte discharge that we observe is reversed in the presence of cis-diltiazem, a drug known to inhibit CNG ion channels in both vertebrate [33] and cnidarian systems [12]. Taken together, our results implicate a role for opsin-based phototransduction in the regulation of cnidocyte discharge in the eyeless cnidarian *H. magnipapillata*.

## Results

### The cellular morphology of cnidocyte battery complexes

In order to establish a morphological framework for comparing gene expression in the battery complexes of our study system *H. magnipapillata*, we explored the cellular composition of these structures using immunohistochemistry and confocal microscopy. Current knowledge of the cellular morphology of cnidocyte-bearing battery complexes is based largely on previous studies that were conducted using electron microscopy (EM) [21,34,35]. A simplified schematic of the hydrozoan battery complex derived from Westfall [36] is shown in Figure 1A. Our results reveal transverse rows of cnidocyte-bearing battery complexes that occur with regular periodicity along the length of the tentacle as described previously for other hydrozoans [37] (Figure 1B). Large stenotele cnidocytes, which function to pierce and envenom prey with harpoon-like tubules, are located at the center of these complexes and are distinguished from other cell types (for example, desmoneme and isorhizal cnidocytes, and sensory neurons) by pronounced α tubulin staining in their cnidocyst capsules [28] and by the presence of large, trigger-like, ciliated cnidocils [37] (Figure 1B-D). Stereocilia, which ensheath the cnidocil and are comprised in part by filamentous actin [24], are revealed in stenotele cnidocytes by phalloidin staining [38] (Figure 1D). Our immunohistochemical studies also show that ganglion cell axonal projections link discrete battery complexes along the proximal/distal axis of the tentacle as previously hypothesized [39] (Figure 1B-D).

### Components of the opsin-mediated phototransduction cascade are co-expressed in cnidocyte battery complexes

**Figure 2:**
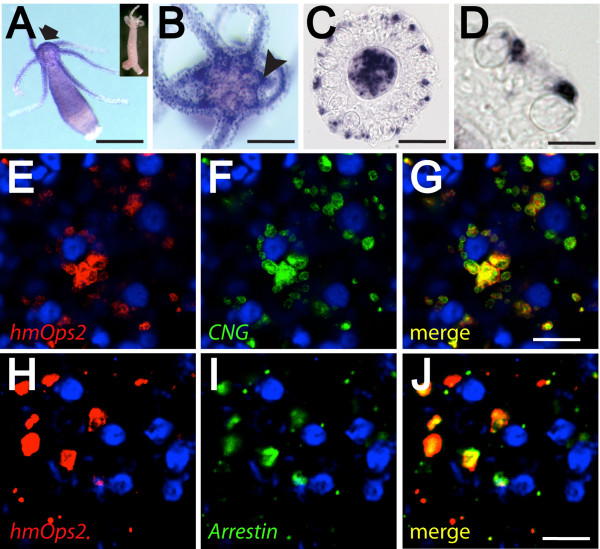
**Studies of gene expression suggest a role for opsin-mediated photosensitivity in the regulation of cnidocyte discharge**. (**A**-**D) **Colorimetric *in situ *hybridization with *HmOps2 *probe. Opsin expression is strongly localized to the hypostome (arrow), tentacles, and the ring-like ganglion (arrow head) that surrounds the mouth (A and B). (C and D) Cryosection of tentacle reveals *HmOps2 *expression in non-cnidocyte cell types. Cnidocytes capsules are clearly visible in these preparations. **(E**-**I) **Confocal fluorescence *in situ *hybridization shows that opsin transcripts co-localize with CNG (E-G) and arrestin (H, I) in battery complexes of the hydra. Blue cells with dark centers are the capsules of stenotele cnidocytes, which stain with DAPI [62]. Signal in (E-I) and (E-G) is located at different focal planes relative to central stenotele cnidocytes. Inset of (A), sense riboprobe negative control. Scale bars in (A) = 1 mm, in (B) = 500 μm, in (C) = 100 μm, in (D) and (E-J) approximately 30 μm.

If phototransduction is involved in the regulation of cnidocyte discharge, we would expect opsin and other components of the cascade to be expressed together in some or all of the battery complex cells described above. In order to test this hypothesis we examined the expression of one hydra opsin, *HmOps2*, using *in situ *hybridization (ISH). This particular opsin was selected for study because its expression pattern has been reported previously [4,12]. Whole mount ISH in *H. magnipapillata *indicated strong *HmOps2 *expression in the ring-like ganglion that encircles the hypostome region and distinct, punctate expression in the tentacles of the animal (Figure 2A and 2B). *HmOps2 *is also expressed diffusely in the body column and in the gastrodermis of the tentacles. Closer inspection, using light microscopy, of cryosections of *HmOps2*-labeled tentacles indicated that ectodermal opsin expression is confined to cells that lack morphological features of cnidocytes, such as the cnidocyst capsules of stenotele, desmoneme and isorhizal cell types, which are clearly visible in these preparations yet lack staining seen in other cells (Figure 2C and 2D). *HmOps2*-expressing-cells likely correspond to sensory-motor neurons described previously from EM studies [35] and immunohistochemistry [39,40].

To further establish the presence of opsin-based phototransduction in battery complex neurons in the tentacles of *H. magnipapillata*, we tested for co-expression between *HmOps2 *and other phototransduction components using florescent ISH (FISH) and confocal microscopy. We first assessed co-expression between *HmOps2 *and CNG. *HmCNG *is the sole CNG gene in the hydra genome and previously has been cloned and analyzed phylogenetically [12]. Our results from confocal optical sections (0.2 μm) corroborate a highly correlated co-expression pattern between *HmOps2 *and *HmCNG *in cells that surround stenotele cnidocytes, which, based on our immunohistochemical studies and much previous work [17], are located at the center of battery complexes (Figure 2D-F).

Next, we tested for co-expression between opsin and arrestin, a gene essential for quenching phototransduction in both vertebrate and insect visual systems [41-43]. We cloned the arrestin orthologue *HmArr1 *from *H. magnipapillata*, which is a member of a clade of non-bilaterian arrestins that is the evolutionary sister to bilaterian β arrestins. Bilaterian β arrestins include visual arrestins known from vertebrate and insect visual systems respectively (Additional file 1). Our results clearly indicate co-expression between *HmOps2 *and *HmArr *in optical sections (0.2 μm) of battery complexes (Figure 2G-I). Together, these findings suggest that a ciliary-type phototransduction cascade comprised in part by opsin, CNG, and arrestin could play a role in the sensory regulation of cnidocyte discharge.

### Cnidocyte discharge is regulated by light

**Figure 3:**
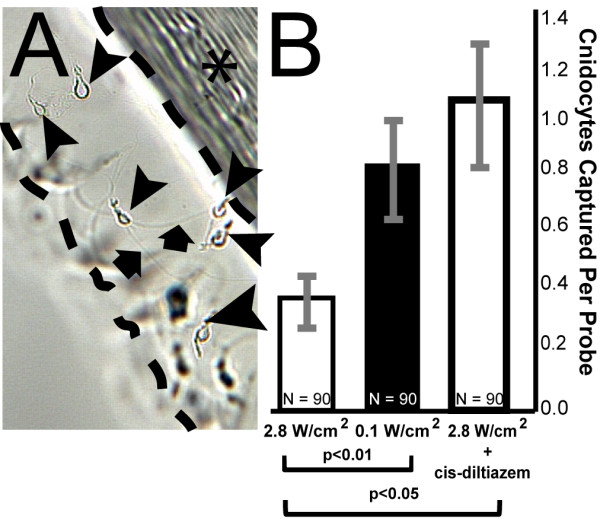
**Behavioral studies of cnidocyte discharge indicate a significant inhibitory effect of light intensity on cnidocyte function**. **(A) **Representative data for cnidocyte discharge assay. In this experiment stenotele cnidocytes were captured using gelatin-coated lengths of monofilament under different experimental conditions. **(B) **Significantly fewer cnidocysts were recovered from assays conducted in bright blue light (470 nm; 2.8 W/cm^2^) than in dim trials (470 nm; 0.1 W/cm^2^). Bright light suppression was ablated by the presence of the CNG inhibitor cis-diltiazem. Area between dashed lines in (A) = gelatin matrix; asterisk = monofilament; arrow heads = stenotele cnidocytes.

In order to test our hypothesis that opsin-based phototransduction functions to modulate cnidocyte discharge, we conducted a series of cnidocyte capture assays under different light conditions. For each assay, animals were acclimated to LED light of different intensities emitted with a very narrow wavelength spectrum peaking at 470 nm. We conducted dim light trials at 0.1 W/cm^2 ^and bright light trials 2.8 W/cm^2 ^(see Methods). Stenotele cnidocytes were captured with probes and counted under light microscopy as per Watson and Hessinger [24]. Our results indicate pronounced effects of the ambient light environment on the propensity for cnidocytes to discharge. Under bright light, the magnitude of cnidocyte discharge was significantly lower than that observed under dim light conditions (Figure 3B).

Because CNG ion channels have been implicated in opsin-mediated phototransduction in cnidarians [10,12] and because transcripts of CNG co-localize with opsin in battery complexes (Figure 2E-G), we tested the efficacy of the CNG ion channel inhibitor cis-diltiazem [33] to ablate the light induced effect on cnidocyte function. Cis-diltiazem (1 μM) reversed the observed reduction of cnidocyte discharge under bright light, as compared to dim light. Further, bright light trials conducted in the presence cis-diltiazem were statistically indistinguishable from control dim light trials performed without the drug (Figure 3B). From this we conclude that environmental information on light intensity acts to regulate cnidocyte discharge and that this property involves CNG function.

## Discussion

Our results provide a window into a previously unknown aspect of cnidarian sensory biology. We demonstrate that opsin-based phototransduction acts to regulate cnidocyte discharge by integrating information from the light environment into a behavioral outcome. Numerous stereotypical photobehaviors ranging from diurnal migration [30], phototaxis [32,44], and the contraction response [32,45] have been described in cnidarians, but no previously published data have demonstrated a role for light information in the regulation of cnidocyte function. We note, however, that an earlier report of unpublished data from studies in the anthozoan *Aptaisia pallida *are consistent with our results [22].

Our immunohistochemical studies of the tentacles of *H. magnipapillata *provide morphological landmarks for correlating the expression of opsin and its biochemical signaling partners with cells that localize to battery complexes. Battery complexes are apparent in our immunohistochemical data as rosettes of nuclei that occur with regular periodicity in transverse rows along the length of the tentacle (Figure 1B). Together with structural features of cnidocytes that are clear in light microscopy (such as the presence of cnidae), we can correlate the expression of opsin to non-stenotele cell types that form the periphery of battery complexes. CNG and arrestin also localize to these opsin-expressing cells as shown by FISH confocal microscopy (Figure 2E-J). We note that the apparent differences in expression pattern between colorimetric (Figure 2A-C) and fluorescent (Figure 2D-F) detection methods likely arise from the higher dynamic range offered by the latter.

Because our colorimetric ISH data show no evidence for opsin expression in any cnidocyte cell type, we conclude that opsin-expressing cells correspond specifically to sensory-motor neurons that were previously noted from EM studies by Westfall [21]. Interestingly, previous EM studies described the presence of pigment granules in these sensory neurons [21], which suggested a possible role in photoreception [46]. These sensory neurons have been reported to form synaptic connections with different cnidocyte cell types within the battery complex, and with ganglion cells that we show here to link separate battery complexes along the proximal/distal axis of the tentacle [39] (Figure 1B). Thus, phototransduction in any battery complex cell type has to potential to regulate firing in adjacent complexes, as has been shown for chemoreception [25].

The functional link that we propose between opsin-mediated phototransduction and cnidocyte discharge is supported by our behavioral studies wherein the effect of light intensity on cnidocyte firing was ablated by the drug cis-diltiazem, a specific inhibitor of CNG [33]. Three observations support our view that cis-diltiazem efficiently perturbs CNG signaling in the context of phototransduction in *H. magnipapillata*. First, all individuals included in cnidocyte firing assays conducted in the presence of the drug were responsive to mechanical stimuli and appeared otherwise normal following experiments (*n *= 90). From this we conclude that non-photoreceptive nervous system function in the hydra is not noticeably affected by cis-diltiazem at experimental concentrations. Second, a single CNG (*HmCNG*) locus is present in the genome sequence of *H. magnipapillata *and the expression of this gene is tightly correlated with that of opsin (Figure 2D-F) [12], thus diminishing the possibility that our drug treatment differentially affected some other unknown, non-phototransductive CNG-based process that indirectly alters the propensity for cnidocyte discharge. Finally, we have previously shown that cis-diltiazem is effective in perturbing the contraction response [12], another light driven behavior in *H. magnipapillata *[7,45]. These factors lead us to conclude that the effect of cis-diltiazem on cnidocyte discharge is specific to CNG and phototransduction.

What might the organismal and ecological relevance of light-regulated cnidocyte discharge be? The integration of light information into cnidocyte firing behavior by opsin-based signaling could make the successful deployment of cnidocytes into prey items or aggressors more likely. Cnidocytes are energetically expensive to maintain and discharge, and they comprise a significant proportion of cells in *H. magnipapillata *[17]. We propose that the accuracy of cnidocyte discharge may be enhanced by the integration of information on ambient light intensity in at least three non-exclusive ways. First, feeding behavior of many freshwater and marine organisms is tuned to diurnal cycles that peak at dusk [30,47]. The significant negative association between cnidocyte discharge and light intensity that we report here could function to reduce the propensity of cnidocytes to discharge during daylight hours, when prey items are scarce, and to arm them at dusk, when productive feeding hours begin. Second, light information in the form of a shadow being cast locally by prey on battery complex sensory neurons could provide another layer of sensory precision, thus enhancing the likelihood that stenotele cnidocytes make contact with and envenom prey items rapidly. This possibility is feasible because the integration of sensory information from the environment into cnidocyte discharge behavior is known to occur within milliseconds [48]. Finally, many cnidarians including *H. magnipapillata *demonstrate positive phototaxis [8,49], which is accomplished by an end-over-end 'somersaulting' behavior. Locomotion of this sort requires non-feeding isorhizal cnidocytes to discharge and adhere to the substrate [31]. One additional possibility is that opsin-mediated inhibition of stenotele and possibly desmoneme discharge, two cnidocyte types that function only in feeding [18] could act to suppress firing in non-locomotory cell types while phototaxis is underway. Our current data cannot discriminate between these possibilities, but provide directions for future research.

## Conclusions

We demonstrate a role for opsin-based phototransduction in the regulation of cnidocyte function. The sensory biology of cnidocyte discharge has intrigued biologists since the 18th century when mechanosensory hair- and trigger-like structures were first hypothesized to actuate cnidocyte function [44,50]. Since that time, chemosensitivity has been added to the repertoire of cnidocyte sensory biology as studies into such cues as amino acids [51], glycoproteins [23], sugars [52], and prey extracts [25] have been demonstrated to alter cnidocyte discharge behavior across a wide phylogenetic range of cnidarians. In each case, the specific sensory receptor genes responsible for these behaviors have remained unknown. Our findings represent the first evidence for a specific sensory receptor gene (for example, opsin) in the regulation of cnidocyte discharge. Future work will seek to understand how light, chemical, and mechanical signal transduction cascades work together to coordinate cnidocyte discharge behavior.

Our results may also have relevance for cnidarian eye evolution, if light modulation of cnidocytes is a general and ancient feature of the phylum. Because immunohistochemical surveys have identified similarities in cnidocyte innervation between representatives of each cnidarian class [53], our finding that light regulates their function in the hydra could extend to other classes as well. Unlike cnidocytes, eyes are found only in some derived cnidarian lineages and may have more recent origins within the phylum [54]. Therefore, we hypothesize that the regulation of cnidocyte discharge by opsin-mediated phototransduction predated this pathway's function in cnidarian eyes. Future studies will investigate the role of light in modulating cnidocyte function in other, non-hydrozoan, lineages and explore the evolutionary history of cnidarian eyes using explicit statistical methods.

## Methods

### Phylogenomics

We conducted BLAST [55] searches for homologs of opsin, CNG, and arrestin from the genome sequences of the non-bilaterian taxa *Amphimedon queenslandica *[56], *Trichoplax adhaerens *[57], *Hydra magnipapillata *[58], and *Nematostela vectensis *[59], together with a taxonomically broad selection of bilaterian genome sequences, using functionally characterized sequences for each gene as queries in custom bioinformatics scripts. We have previously described the phylogenetic relationships and placement of cnidarian opsin [4] and CNG [12] using these methods and a global arrestin phylogeny has been described elsewhere [60]. The details of our methods and a phylogenetic analysis aimed specifically at the β arrestin subclass, which includes bilaterian visual arrestins, are provided in Additional files 1 and 2.

### Immunohistochemistry and *in situ *hybridization

For immunohistochemistry and *in situ *hybridization animals were starved for two days prior to staining. Animals were relaxed in 2% urethane (Sigma) in hydra medium (HM; 1.0 mM CaCl2, 1.5 mM NaHCO3, 0.1 mM MgCl2, 0.008 mM MgSO4, 0.03 mM KNO3; pH 8.0) and fixed overnight at 4°C in 4% paraformaldehyde (Sigma) in HM.

For immunohistochemistry, animals were washed five times for 5 min in PBST (3.2 mM Na2HPO4, 0.5 mM KH2PO4, 1.3 mM KCl, 135 mM NaCl, 0.1% Tween 20, pH 7.4) and blocked for 2 h in PBST + 20% normal goat serum (NGS; Sigma) at room temperature. Animals were then incubated with primary antibody, anti-acetylated α tubulin (1:500; Sigma), in blocking solution overnight at 4°C. Following primary antibody, animals were washed five times for 5 min in PBST and blocked as before. Secondary antibody, Cy2-conjugated anti mouse Ig (Jackson), was added over night at 4°C in blocking reagent. Samples were then washed five times for 5 min in PBST and a solution containing 1:1000 of DAPI stock (Molecular Probes) and 1:1000 Alexa Fluor 488-labeled phalloidin stock (Invitrogen) in PBST was added for 1 h. Samples were then washed five times for 5 min and mounted in glycerol.

For *in situ *hybridization, all loci were cloned based on publicly available EST sequences. *HmOps2 *corresponds to CV151648, *HmCNG *corresponds to DT606755 (a single-copy gene) and *HmArr *corresponds to CV464909. FISH confocal microscopy and RNA probe construction for *HmOps2, HmCNG*, and *HmArr *were conducted as previously reported [12].

### Animal culture and cnidocyte capture assay

*Hydra magnipapillata *(strain 105) was cultured using standard methods in HM [61]. Our cnidocyte capture assay was based on the method of Watson and Hessinger [24]. Lengths of fishing line (10 cm) were dipped twice in 20% gelatin (Knox) that had been dissolved in HM and heated to 65°C. Gelatin concentration was optimized in preliminary trials. Polymerized probes were used within 1 h of fabrication. For cnidocyte capture trials, we placed healthy and responsive animals in either dim blue light (0.1 W/cm^2^) or bright blue light (2.8 W/cm^2^) for 6 h prior to probing. In each case, light came from a blue (470 nm peak) LED array (SuperBright LEDs, part # E27-B24) placed at different distances from the animals to adjust brightness. To measure light intensity, we used a Jaz ULM spectrometer (Ocean Optics, Dunedin, FL, USA). We integrated intensity measurements between 435 nm and 515 nm wavelengths, using an integration time of 0.1 s. Ten individuals were placed into a test arena and animals were probed once at the distal tips of tentacles for approximately 1 s before the probe was withdrawn. The distal 1 cm of the probes were cut and mounted in glycerol. Cnidocytes that were collected in the probes were counted by light microscopy at 40×. The same procedure was used during trials with the CNG inhibitor cis-diltiazem (1 μM in HM; Sigma). Owing to the harpoon-like structure of discharged stenotele cnidocils, discharge data on these cell types are preferentially recovered.

Our experimental design first tested for differences between trials under bright and dim light, and then tested for differences between trials under bright light and bright light + cis-diltiazem. Data from 90 replicates from each experimental condition passed a D'Agostino & Pearson omnibus test of normality. Data were then compared using unpaired t-tests in Prism 5 (GraphPad).

## Abbreviations

CNG: cyclic nucleotide gated ion channel; HM: hydra medium; ISH: *in situ *hybridization

## Competing interests

The authors declare that they have no competing interests.

## Authors' contributions

DCP conceived and designed the study, carried out some of the behavioral assays, analyzed the data, and drafted the manuscript. CRF contributed to the design and implementation of the majority of behavioral assays. THO contributed to the design of the study and drafted the manuscript. All authors have read and approved the final manuscript.

## Supplementary Material

Additional file 1 **Phylogenetic tree of metazoan arrestins**. Phylogenetic analyses of metazoan β arrestin genes. ML topology shown. Nodal support is given by bootstrap percentages/posterior probability. Support values below 50 are not shown. Dashes = 100% for bootstrap support and 1.0 for posterior probability. Click here for file

Additional file 2 **Supplementary methods**. Click here for file

## References

[B1] Hardie RC Raghu P Visual transduction in Drosophila Nature 2001 413 186 193 10.1038/35093002 11557987

[B2] Matulef K Zagotta WN Cyclic nucleotide-gated ion channels Annu Rev Cell Dev Biol 2003 19 23 44 10.1146/annurev.cellbio.19.110701.154854 14570562

[B3] Venkatachalam K Montell C TRP channels Annu Rev Biochem 2007 76 387 417 10.1146/annurev.biochem.75.103004.142819 17579562 PMC4196875

[B4] Plachetzki DC Degnan BM Oakley TH The origins of novel protein interactions during animal opsin evolution PLoS One 2007 2 e1054 10.1371/journal.pone.0001054 17940617 PMC2013938

[B5] Suga H Schmid V Gehring WJ Evolution and functional diversity of jellyfish opsins Curr Biol 2008 18 51 55 10.1016/j.cub.2007.11.059 18160295

[B6] Porter ML Blasic JR Bok MJ Cameron EG Pringle T Cronin TW Robinson PR Shedding new light on opsin evolution Proc Biol Sci 2012 279 3 14 10.1098/rspb.2011.1819 22012981 PMC3223661

[B7] Passano LM McCullough CB The light response and the rhythmic potentials of hydra Proc Natl Acad Sci USA 1962 48 1376 1382 10.1073/pnas.48.8.1376 16590985 PMC220962

[B8] Singer RH Rushforth NB Burnett AL The photodynamic action of light on hydra J Exp Zool 1963 154 169 173 10.1002/jez.1401540204 14085414

[B9] Musio C Santillo S Taddei-Ferretti C Robles LJ Vismara R Barsanti L Gualtieri P First identification and localization of a visual pigment in Hydra (Cnidaria, Hydrozoa) J Comp Physiol [A] 2001 187 79 81 10.1007/s003590100180 11318381

[B10] Koyanagi M Takano K Tsukamoto H Ohtsu K Tokunaga F Terakita A Jellyfish vision starts with cAMP signaling mediated by opsin-G(s) cascade Proc Natl Acad Sci USA 2008 105 15576 15580 10.1073/pnas.0806215105 18832159 PMC2563118

[B11] Kozmik Z Ruzickova J Jonasova K Matsumoto Y Vopalensky P Kozmikova I Strnad H Kawamura S Piatigorsky J Paces V Vlcek C Assembly of the cnidarian camera-type eye from vertebrate-like components Proc Natl Acad Sci USA 2008 105 8989 8993 10.1073/pnas.0800388105 18577593 PMC2449352

[B12] Plachetzki DC Fong CR Oakley TH The evolution of phototransduction from an ancestral cyclic nucleotide gated pathway Proc Biol Sci 2010 277 1963 1969 10.1098/rspb.2009.1797 20219739 PMC2880087

[B13] Wilden U Kuhn H Light-dependent phosphorylation of rhodopsin: number of phosphorylation sites Biochemistry 1982 21 3014 3022 10.1021/bi00541a032 6980670

[B14] Krupnick JG Gurevich VV Schepers T Hamm HE Benovic JL Arrestin-rhodopsin interaction. Multi-site binding delineated by peptide inhibition J Biol Chem 1994 269 3226 3232 8106358

[B15] Xu J Dodd RL Makino CL Simon MI Baylor DA Chen J Prolonged photoresponses in transgenic mouse rods lacking arrestin Nature 1997 389 505 509 10.1038/39068 9333241

[B16] Dolph PJ Ranganathan R Colley NJ Hardy RW Socolich M Zuker CS Arrestin function in inactivation of G protein-coupled receptor rhodopsin in vivo Science 1993 260 1910 1916 10.1126/science.8316831 8316831

[B17] Hessinger DA Lenhoff HM The Biology of Nematocysts 1988 San Diego, CA: Academic Press, Inc.

[B18] Kass-Simon GSAA The behavioral and developmental physiology of nematocysts Can J Zool 2002 80 1772 1794 10.1139/z02-135

[B19] Mariscal RN Muscatine L, Lenhoff HM Nematocysts Colenterate Biology: Reviews and New Perspectives 1974 New York: Academic Press 129 166

[B20] Hyman L The Invertebrates: Protozoa through Ctenophora 1940 New York: McGraw-Hill

[B21] Westfall JA Kinnamon JC A second sensory--motor--interneuron with neurosecretory granules in Hydra J Neurocytol 1978 7 365 379 10.1007/BF01176999 207827

[B22] Thorington GU Hessinger DA Hessinger DA, Lenhoff HM Control of discharge: factors affecting discharge of cnidae The Biology of Nematocyts 1988 San Diego, CA: Academic Press 233 254

[B23] Watson G Hessinger D Antagonistic frequency tuning of hair bundles by different chemoreceptors regulates nematocyst discharge J Exp Biol 1994 187 57 73 9317331 10.1242/jeb.187.1.57

[B24] Watson GM Hessinger DA Cnidocyte mechanoreceptors are tuned to the movements of swimming prey by chemoreceptors Science 1989 243 1589 1591 10.1126/science.2564698 2564698

[B25] Price RB Anderson PA Chemosensory pathways in the capitate tentacles of the hydroid Cladonema Invert Neurosci 2006 6 23 32 10.1007/s10158-005-0015-6 16421749

[B26] Hwang JS Ohyanagi H Hayakawa S Osato N Nishimiya-Fujisawa C Ikeo K David CN Fujisawa T Gojobori T The evolutionary emergence of cell type-specific genes inferred from the gene expression analysis of Hydra Proc Natl Acad Sci USA 2007 104 14735 14740 10.1073/pnas.0703331104 17766437 PMC1963347

[B27] Milde S Hemmrich G Anton-Erxleben F Khalturin K Wittlieb J Bosch TC Characterization of taxonomically restricted genes in a phylum-restricted cell type Genome Biol 2009 10 R8 19161630 10.1186/gb-2009-10-1-r8 PMC2687796

[B28] Hwang JS Takaku Y Momose T Adamczyk P Ozbek S Ikeo K Khalturin K Hemmrich G Bosch TC Holstein TW David CN Gojobori T Nematogalectin, a nematocyst protein with GlyXY and galectin domains, demonstrates nematocyte-specific alternative splicing in Hydra Proc Natl Acad Sci USA 2010 107 18539 18544 10.1073/pnas.1003256107 20937891 PMC2972925

[B29] Pantin CFA The excitation of nematocysts J Exp Biol 1942 19 294 310

[B30] Mackie GO Colenterate organs Mar Freshw Behav Physiol 1999 32 113 127 10.1080/10236249909379043

[B31] Ewer RF On the functions and mode of action of the nematocytes of hydra Proc Zool Soc Lond 1947 117 365 376

[B32] Rushforth NB Corning WC, Dyal JA, Willows AOD Behavioral modifications in Colenterates Invertebrate Learning 1973 1 New York: Plenum 123 269

[B33] Haynes LW Block of the cyclic GMP-gated channel of vertebrate rod and cone photoreceptors by l-cis-diltiazem J Gen Physiol 1992 100 783 801 10.1085/jgp.100.5.783 1282145 PMC2229114

[B34] Slautterback DB Cytoplasmic microtubules. I. Hydra J Cell Biol 1963 18 367 388 10.1083/jcb.18.2.367 14079495 PMC2106295

[B35] Westfall JA Ultrastructural evidence for a granule-containing sensory-motor-interneuron in Hydra littoralis J Ultrastruct Res 1973 42 268 282 10.1016/S0022-5320(73)90055-5 4702922

[B36] Westfall J Hessinger D, Lenhoff H Presumed neuronematocyte synapse and possible pathways controlling discharge of a battery of nematocysts in hydra The Biology of Nematocytes 1988 San Diego, CA: Academic Press, Inc. 41 47

[B37] Holstein T Hausmann K Hessinger DA, Lenhoff HM The cnidocil apparatus of hydrozoans: a progenitor of higher metazoan mechanoreceptors? The biology of nematocytes 1988 San Deigo, CA: Academic Press, Inc. 53 73

[B38] Wood RL Novak PL The anchoring of nematocysts and nematocytes in the tentacles of hydra J Ultrastruct Res 1982 81 104 116 10.1016/S0022-5320(82)90044-2 6890581

[B39] Yu SM Westfall JA Dunne JF Light and electron microscopic localization of a monoclonal antibody in neurons in situ in the head region of Hydra J Morphol 1985 184 183 193 10.1002/jmor.1051840208 3989866

[B40] Hobmayer E Holstein TW David CN Tentacle morphogenesis in hydra. II. Formation of a complex between a sensory nerve-cell and a battery cell Development 1990 109 897 904

[B41] Krupnick JG Gurevich VV Benovic JL Mechanism of quenching of phototransduction. Binding competition between arrestin and transducin for phosphorhodopsin J Biol Chem 1997 272 18125 18131 10.1074/jbc.272.29.18125 9218446

[B42] Xu TR Baillie GS Bhari N Houslay TM Pitt AM Adams DR Kolch W Houslay MD Milligan G Mutations of beta-arrestin 2 that limit self-association also interfere with interactions with the beta2-adrenoceptor and the ERK1/2 MAPKs: implications for beta2-adrenoceptor signalling via the ERK1/2 MAPKs Biochem J 2008 413 51 60 10.1042/BJ20080685 18435604

[B43] Dolph PJ Arrestin: roles in the life and death of retinal neurons Neuroscientist 2002 8 347 355 10.1177/107385840200800410 12194503

[B44] Trembley A Memoires pour srvir a l'histore d'un genre de polypes d'eau douce a brasen forme de cornes 1744 Leyden: J & A Verbeck

[B45] Passano LM McCullough CB Pacemaker hierarchies controlling the behaviour of hydras Nature 1963 199 1174 1175 10.1038/1991174a0 14072038

[B46] Eakin RM Westfall JA Fine structure of photoreceptors in the hydromedusan, polyorchis penicillatus Proc Natl Acad Sci USA 1962 48 826 833 10.1073/pnas.48.5.826 16590949 PMC220861

[B47] Slobodkin LB Bossert PE Thorp HA, Covich AP Cnidaria Ecology and classification of North American freshwater invertebrates 2001 New York: Academic Press Inc 135 154

[B48] Nuchter T Benoit M Engel U Ozbek S Holstein TW Nanosecond-scale kinetics of nematocyst discharge Curr Biol 2006 16 R316 318 10.1016/j.cub.2006.03.089 16682335

[B49] Pardy RL Mackie GO Aspects of light in the biology of green hydra Coelenterate ecology and behavior 1976 New York: Plenum Press 401 408

[B50] Baker H Some observations on the polype dried Phil Trans 1744 42 616 619

[B51] Watson GM Roberts J Chemoreceptor-mediated polymerization and depolymerization of actin in hair bundles of sea anemones Cell Motil Cytoskeleton 1995 30 208 220 10.1002/cm.970300305 7758137

[B52] Watson GM Hessinger DA Receptors for N-acetylated sugars may stimulate adenylate cyclase to sensitize and tune mechanoreceptors involved in triggering nematocyst discharge Exp Cell Res 1992 198 8 16 10.1016/0014-4827(92)90142-U 1309195

[B53] Anderson PA Thompson LF Moneypenny CG Evidence for a common pattern of peptidergic innervation of cnidocytes Biol Bull 2004 207 141 146 10.2307/1543588 15501855

[B54] Salvini-Plawen LV Mayr E On the evolution of photoreceptors and eyes 1977 10 New York: Plenum Press

[B55] Altschul SF Madden TL Schaffer AA Zhang J Zhang Z Miller W Lipman DJ Gapped BLAST and PSI-BLAST: a new generation of protein database search programs Nucleic Acids Res 1997 25 3389 3402 10.1093/nar/25.17.3389 9254694 PMC146917

[B56] Srivastava M Simakov O Chapman J Fahey B Gauthier ME Mitros T Richards GS Conaco C Dacre M Hellsten U Larroux C Putnam NH Stanke M Adamska M Darling A Degnan SM Oakley TH Plachetzki DC Zhai Y Adamski M Calcino A Cummins SF Goodstein DM Harris C Jackson DJ Leys SP Shu S Woodcroft BJ Vervoort M Kosik KS The Amphimedon queenslandica genome and the evolution of animal complexity Nature 2010 466 720 726 10.1038/nature09201 20686567 PMC3130542

[B57] Srivastava M Begovic E Chapman J Putnam NH Hellsten U Kawashima T Kuo A Mitros T Salamov A Carpenter ML Signorovitch AY Moreno MA Kamm K Grimwood J Schmutz J Shapiro H Grigoriev IV Buss LW Schierwater B Dellaporta SL Rokhsar DS The Trichoplax genome and the nature of placozoans Nature 2008 454 955 960 10.1038/nature07191 18719581

[B58] Chapman JA Kirkness EF Simakov O Hampson SE Mitros T Weinmaier T Rattei T Balasubramanian PG Borman J Busam D Disbennett K Pfannkoch C Sumin N Sutton GG Viswanathan LD Walenz B Goodstein DM Hellsten U Kawashima T Prochnik SE Putnam NH Shu S Blumberg B Dana CE Gee L Kibler DF Law L Lindgens D Martinez DE Peng J The dynamic genome of Hydra Nature 2010 464 592 596 10.1038/nature08830 20228792 PMC4479502

[B59] Putnam NH Srivastava M Hellsten U Dirks B Chapman J Salamov A Terry A Shapiro H Lindquist E Kapitonov VV Jurka J Genikhovich G Grigoriev IV Lucas SM Steele RE Finnerty JR Technau U Martindale MQ Rokhsar DS Sea anemone genome reveals ancestral eumetazoan gene repertoire and genomic organization Science 2007 317 86 94 10.1126/science.1139158 17615350

[B60] Alvarez CE Robison K Gilbert W Novel Gq alpha isoform is a candidate transducer of rhodopsin signaling in a Drosophila testes-autonomous pacemaker Proc Natl Acad Sci USA 1996 93 12278 12282 10.1073/pnas.93.22.12278 8901571 PMC37981

[B61] Lenhoff H Lenhoff H Water, Culture Solutions and Buffers Hydra Research Methods 1982 New York: Plenum 19 28

[B62] Szczepanek S Cikala M David CN Poly-gamma-glutamate synthesis during formation of nematocyst capsules in Hydra J Cell Sci 2002 115 745 751 11865030 10.1242/jcs.115.4.745

