# Supplementary figures and images for: Cnidocyte discharge is regulated by light and opsin-mediated phototransduction
Source: BMC Biol. 2012 Mar 5;10:17. doi: 10.1186/1741-7007-10-17 (PMC3329406; doi:10.1186/1741-7007-10-17)

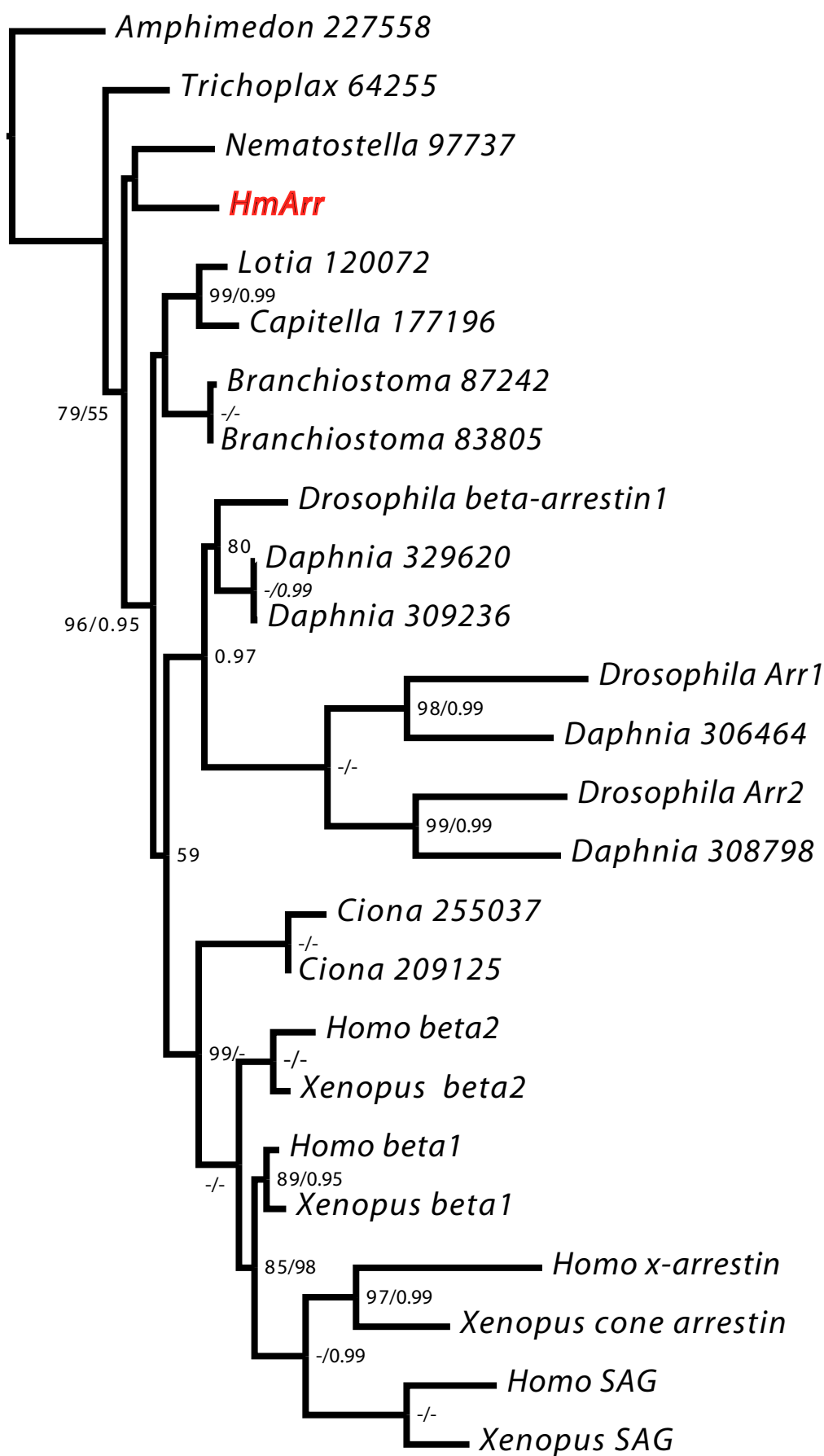

0.2

Supplement: Additional file 1 — Phylogenetic tree of metazoan arrestins. Phylogenetic analyses of metazoan β arrestin genes. ML topology shown. Nodal support is given by bootstrap percentages/posterior probability. Support values below 50 are not shown. Dashes = 100% for bootstrap support and 1.0 for posterior probability. [file 1741-7007-10-17-S1.PDF]
