# Supplementary material for: Cnidocyte discharge is regulated by light and opsin-mediated phototransduction
Source: BMC Biol. 2012 Mar 5;10:17. doi: 10.1186/1741-7007-10-17 (PMC3329406; doi:10.1186/1741-7007-10-17)
Supplement: Additional file 2 — Supplementary methods. [file 1741-7007-10-17-S2.PDF]

Plachetzki et al,

Supplemental Methods

For arrestin phylogeny, we used an in house data assembly pipeline that implements BLAST (1) under default parameters to search the draft genome sequences of the following bilaterian and non-bilaterian taxa: *Amphimedon queenslandica*, *Branchiostoma floridae*, *Capitella teleta*, *Ciona intestinalis*, *Daphnia pulex*, *Drosophila melanogaster*, *Homo sapiens*, *Hydra magnipapillata*, *Lotia gigantae*, *Nematostella vectensis* and *Trichoplax adhaerens* and *Xenopus laevis*. For BLAST searches we used the following, previously characterized sequences as queries: *H. sapiens* SAG arrestin (NP_000532.2) and *D. melanogaster* Arr1 (NP_476681). All sequences were obtained from GenBank. The ten best scoring sequences from each genome sequence were retained. The resulting sequences were analyzed using Blast2GO and spurious sequences that did not blast to arrestin homologues were removed. This dataset was aligned using MAFTT (2) and the best-fit model for molecular evolution, JTT, was ascertained using PROTTEST (3). A first round of phylogenetic analysis was done using RaxML (4) under JTT. The sequences that comprised the α arrestin clade, which do not contain visual arrestins, were removed by referring to a previous analysis of arrestin phylogeny (5). This reduced dataset of metazoan β arrestins was aligned as before and analyzed with a second round of phylogenetic analyses which included fast bootstrapping in RaxML (4) under JTT, and Bayesian analyses in phylobayes (6) under the CAT model. We rooted these trees with the single β sequence that was recovered from the genome of the sponge, *Amphemedon queenslandica*.

1. Altschul SF*, et al.* (1997) Gapped BLAST and PSI-BLAST: a new generation of protein database search programs. *Nucleic Acids Res* 25(17):3389-3402.
2. Katoh K, Kuma K, Toh H, & Miyata T (2005) MAFFT version 5: improvement in accuracy of multiple sequence alignment. (Translated from eng) *Nucleic Acids Res* 33(2):511-518 21(9):2104-2105.
4. Stamatakis A, Ludwig T, & Meier H (2005) RAxML-III: a fast program for maximum likelihood-based inference of large phylogenetic trees. (Translated from eng) *Bioinformatics* 21(4):456-463 .
5. Alvarez CE, Robison K, & Gilbert W (1996) Novel Gq alpha isoform is a candidate transducer of rhodopsin signaling in a Drosophila testes-autonomous pacemaker. *Proc Natl Acad Sci U S A* 93(22):12278-12282.
6. Lartillot N & Philippe H (2004) A Bayesian mixture model for across-site heterogeneities in the amino-acid replacement process. (Translated from eng) *Mol Biol Evol* 21(6):1095-1109 .

Fig S1. Phylogenetic analyses of metazoan β arrestin genes. ML topology shown. Nodal support is given by bootstrap percentages / and posterior probability. Support values below 50 are not shown. Dashes = 100% for bootstrap support and 1.0 for posterior probability.
